# Association between self-objectification and emotional eating among female college students: the sequential indirect role of social comparison and body satisfaction

**DOI:** 10.3389/fpsyg.2026.1803124

**Published:** 2026-09-03

**Authors:** Yuqiu Li, Guoping Wu, Qing Dong, Zhe Dong, Xiaorui Wang, Zhenhua Li

**Affiliations:** 1 The Second People's Hospital of Chuxiong Yi Autonomous Prefecture, Chuxiong, Yunnan, China; 2 Peking University HuiLongGuan Clinical Medical School, Beijing Huilongguan Hospital, Beijing, China

**Keywords:** body satisfaction, emotional eating, female college students, self-objectification, social comparison

## Abstract

**Background:**

The college years represent a formative period for body image development in young women, and female college students (FCS) have an elevated risk of disordered eating behaviors, including emotional eating.

**Objective:**

This cross-sectional study explores the relationship between self-objectification and emotional eating in FCS and examines the mediating role of social comparison and body satisfaction.

**Methods:**

A total of 820 FCS from two comprehensive universities in Yunnan, China, were enrolled. Data were collected in June 2023 using self-report scales: the Trait Self-Objectification Questionnaire, Emotional Eating Subscale of the Dutch Eating Behavior Questionnaire, Physical Appearance Comparison Scale, and Body Image States Scale. Correlation and mediation analyses were conducted.

**Results:**

Self-objectification, emotional eating, and social comparison exhibit significant positive correlations with each other (*r*: 0.11–0.33, *p* < 0.01). Body satisfaction shows significant negative correlations with self-objectification, emotional eating, and social comparison (*r*: −0.28 – −0.17, *p* < 0.01). Social comparison and body satisfaction fully accounted for the association between self-objectification and emotional eating, with effect sizes of 49.90 and 20.34%, respectively.

**Conclusion:**

Self-objectification can indirectly affect emotional eating through social comparison and body satisfaction.

## 1 Introduction

The rapid proliferation of mass media, social networking platforms, and global fashion culture has amplified sociocultural norms that prioritize women’s physical appearance as a primary marker of social worth. Mounting cross-cultural evidence documents that this systemic shift has driven a steep, global rise in the prevalence of self-objectification among women (Chang et al., 2026; Saunders and Eaton, 2018; Tang et al., 2024). First formalized by Fredrickson and Roberts (1997), objectification theory conceptualizes self-objectification as a maladaptive self-perceptual style: when individuals internalize external, appearance-focused societal standards, they come to view their bodies primarily as objects to be evaluated by others. Subsequent work by Daniels et al. (2020) further clarifies that women with elevated trait self-objectification consistently prioritize outward appearance over physical functioning, internal capacities, and non-physical traits, while engaging in persistent, excessive preoccupation with their looks and chronic body surveillance.

research has confirmed that self-objectification predicts a range of adverse mental health outcomes, including anxiety, depression, diminished flow experiences, sexual dysfunction and alcohol misuse (Noll and Fredrickson, 1998; Carretta and Szymanski, 2022; Kahalon and Klein, 2025). Existing evidence indicates robust covariation between self-objectification and disordered eating behaviors, which are frequently identified as prominent correlates of self-objectification (Sarda et al., 2025). Despite this well-established link, the extant literature overwhelmingly focuses on restrictive eating behaviors in the context of self-objectification (Niu et al., 2020; Ma and Lan, 2022; Hu et al., 2025). By contrast, emotional eating—a distinct phenotype driven by negative affect rather than physiological hunger—has received considerably less empirical attention within the objectification theory framework. Among the few studies that have examined emotional eating, most have been conducted in Western adult or clinical populations, leaving the applicability of these findings to non-Western, non-clinical young female samples largely unknown.

### 1.1 Relationship between self-objectification and emotional eating

Emotional eating is defined as an acquired emotion regulation strategy where individuals use food to cope with negative emotional distress, independent of physiological hunger (Evers et al., 2010; Li et al., 2018; Zhao et al., 2022). It is a core clinical dimension of eating disorders and a well-established risk factor for obesity, anorexia nervosa, and bulimia nervosa (Ricca et al., 2012).

Two well-established theoretical frameworks explain emotional eating. First, emotion regulation theory posits that individuals with maladaptive coping strategies use overeating to mitigate negative affect. Second, escape theory holds that eating serves to disengage individuals from aversive self-evaluations and acute stressors (Nederkoorn et al., 2006). Emotional eaters exhibit heightened vulnerability to stress. When confronted with psychological stressors, they experience elevated cortisol levels. This response further perpetuates problematic eating patterns, creating a vicious cycle of emotional eating (Chang et al., 2022). Therefore, negative affect is universally acknowledged as the core triggering factor for emotional eating (Dakanalis et al., 2023; Muha et al., 2024).

Given that female college students (FCS) are in a critical developmental period for body image formation and are particularly vulnerable to maladaptive eating patterns (Tozun et al., 2010; van Mierlo et al., 2021), understanding the mechanisms that link self-objectification to emotional eating in this specific population is of both theoretical and practical importance.

### 1.2 The mediating role of social comparison and body satisfaction

There exists a consistent correlational link between self-objectification and body-related negative affect (Saunders et al., 2024). researchers have yet to clarify whether it drives emotional eating directly, or indirectly through intermediate cognitive and affective pathways.

Festinger (1954) first established social comparison theory, which conceptualizes the innate human tendency to evaluate one’s own attributes, abilities and social standing through comparative judgments with referent others. In contemporary appearance-focused society, this process has expanded to center heavily on physical appearance. Previous studies have shown that higher levels of appearance-based social comparison are associated with increased thinness-seeking tendencies and disordered eating behaviors (Cheng, 2020; Gilbert and Meyer, 2003), and patients with eating disorders exhibit more frequent social comparison (Corning and Krumm, 2006). Furthermore, women with high self-objectification habitually monitor their appearance and engage in more frequent social comparisons with peers (Yang et al., 2020). which may generate negative emotions and subsequently trigger emotional eating. This suggests that social comparison may mediate the relationship between self-objectification and emotional eating.

Body satisfaction refers to the degree of positive perception of one’s physical appearance and the perceived discrepancy between actual and ideal body. Body dissatisfaction is associated with maladaptive weight-control behaviors and elevated risks of depressive symptoms and disordered eating (van Mierlo et al., 2021; Gitimu et al., 2016). Self-objectification is significantly negatively correlated with body satisfaction (Zhang and Hu, 2021), and body dissatisfaction is a well-documented proximal correlate and putative risk factor for anorexia nervosa, bulimia nervosa, and emotional eating (Lindner et al., 2012; Mölbert et al., 2017).

Integrating these two perspectives, social comparison and body satisfaction may function as both parallel and sequential mediators. Frequent appearance comparisons reduce body satisfaction by highlighting gaps between one’s own body and societal beauty standards (Rancourt et al., 2016). This creates a sequential pathway from self-objectification to social comparison to body satisfaction to emotional eating.

### 1.3 The influences of body mass index (BMI)

Multiple studies demonstrate that higher BMI is associated with greater body shame and elevated levels of trait self-objectification (Slevec and Tiggemann, 2011; Schaefer and Thompson, 2018; Cella et al., 2020). Building on these findings, we infer that BMI and self-objectification may interact to predict body satisfaction outcomes and eating behaviors; accordingly, we include BMI as a covariate in all statistical analyses to isolate the unique effects of our core study variables.

### 1.4 The present study

Taken together, prior studies have verified a stable link between self-objectification and disordered eating, yet three limitations remain. First, most research focuses on restrictive eating instead of emotion-triggered emotional eating. Second, existing evidence mainly comes from Western samples and young women in economically developed areas of China, while inland Chinese populations are understudied. Third, few studies have clarified the complete serial cognitive-affective pathways connecting self-objectification to disordered eating.

Given these understudied areas, this study proposes two core aims. First, we explore the cross-sectional association between self-objectification and emotional eating among female college students in inland Yunnan, China. Second, we test the parallel and sequential mediating roles of appearance social comparison and body satisfaction in this association, adjusting for BMI and sociodemographic covariates. This research aims to broaden the application of objectification theory to emotional eating and provide practical evidence for campus mental health interventions targeting disordered eating in young women. We put forward four hypotheses accordingly:

*Hypothesis 1*: Self-objectification is significantly positively correlated with emotional eating in FCS.

*Hypothesis 2*: Social comparison mediates the relationship between self-objectification and emotional eating.

*Hypothesis 3*: Body satisfaction mediates the relationship between self-objectification and emotional eating.

*Hypothesis 4*: Social comparison and body satisfaction exert a sequential indirect effect between self-objectification and emotional eating.

By testing these hypotheses, this study makes three key contributions. Theoretically, it extends objectification theory to the less-studied field of emotional eating by distinguishing parallel and serial mediation through cognitive (social comparison) and affective (body satisfaction) paths. Empirically, we offset the sampling bias of previous literature with inland Chinese college student data, supplementing findings from Western and China’s developed regions. Practically, the identified psychological pathways offer adjustable intervention targets to lower emotional eating risks and guide mental health prevention for FCS.

## 2 Methods

### 2.1 Study design

This study employed a cross-sectional design to examine the associations between self-objectification, appearance-based social comparison, body satisfaction, and emotional eating among female college students. All procedures were approved by the Ethics Committee of the Second People’s Hospital of Chuxiong Yi Autonomous Prefecture [(2025) Research No. 12]. The target range of 800–850 valid participants was pre-specified based on three considerations: (1) widely observed patterns in research on self-objectification and disordered eating, whereby cross-sectional mediation studies often recruit between 600 and 1,000 participants (Schaefer and Thompson, 2018); (2) methodological guidelines for serial mediation analysis, which recommend a minimum sample size of 500 to reliably detect small-to-medium indirect effects at acceptable statistical power; (3) an estimated 90% valid response rate for on-site paper-and-pencil surveys. Data collection was conducted in June 2023 and concluded once this target was met.

A post-hoc power analysis was performed in G*Power 3.1 using the full regression model predicting emotional eating (*f*^2^ = 0.075, R^2^ = 0.07, 8 predictors including covariates). At a two-tailed *α* = 0.05, the final sample of 820 achieved statistical power > 0.99, confirming sufficient power for all core analyses.

### 2.2 Participants

Female college students (FCS) from two comprehensive universities in Yunnan, China, were recruited via convenience sampling. Trained psychology master’s students distributed flyers and provided verbal briefings in campus libraries and dormitories. The briefings explained the study purpose, questionnaire content, 15–20 min completion time, and fully voluntary participation. All participants completed paper questionnaires in quiet study rooms, with immediate on-site collection. Inclusion criteria: (1) Officially enrolled FCS at the two universities; (2) Able to complete the questionnaire independently. Exclusion criteria: (1) Missing >15% of items or key variables; (2) Obvious response bias (e.g., identical answers for all items, contradictory reverse-coded responses).

A total of 910 questionnaires were distributed. After excluding invalid responses, 820 valid questionnaires were retained, yielding an effective response rate of 90.01%.

### 2.3 Measures

Body Mass Index (BMI) is an objective quantitative indicator based on weight and height, commonly used to assess an individual’s weight status and degree of obesity. It is one of the most frequently employed anthropometric indicators in epidemiological surveys, clinical research, and public health fields. In this study, BMI was calculated from self-reported height (in centimeters) and weight (in kilograms).

Emotional eating behavior was measured using the Emotional Eating Sub questionnaire from the Dutch Eating Behavior Questionnaire (DEBQ) developed by Li et al. (2018). Items such as “Do you crave food when you feel nervous, worried, or anxious?” were used to assess participants’ levels of emotional eating. This subscale consists of 13 items rated on a five-point Likert scale, where 1 indicates “never” and 5 indicates “always.” Higher scores indicated a greater tendency toward emotional eating. The Cronbach’s *α* coefficient for this study was 0.94.

Self-objectification was assessed using the Trait Self-Objectification Questionnaire revised by Yu (2016) for Chinese college students. The original scale was developed based on objectification theory, and the Chinese version has been validated to have good reliability and validity in Chinese FCS populations. The scale consists of 10 body-related descriptors, including 5 appearance-related items and 5 physical competence-related items. Participants were asked to rank the 10 descriptors from 9 (highest importance) to 0 (lowest importance) according to their level of concern. The total score was calculated by subtracting the sum of physical competence items from the sum of appearance items, with higher scores indicating higher levels of self-objectification.

Social comparison was assessed using the Chinese version of the Physical Appearance Comparison Scale (Chen et al., 2007), featuring items such as “In social situations, I compare my body with others’ bodies.” This tool evaluates social comparison traits related to physical appearance. The questionnaire comprised five items, requiring participants to rate the extent to which each description applied to them on a scale from 1 (“Never”) to 5 (“Always”). Higher scores indicated more frequent social comparison. The Cronbach’s *α* coefficient for this study was 0.81.

Body satisfaction was assessed using the Body Image States Scale by Cash et al. (2002), comprising six items. Responses ranged from “very dissatisfied” to “very satisfied” on a 7-point scale, with higher scores indicating greater current body satisfaction. The Cronbach’s α coefficient for this study was 0.89.

### 2.4 Statistical analysis

All statistical analyses were performed using IBM® SPSS® Statistics 25.0 with the PROCESS macro Version 3.3 (Hayes, 2013). A two-tailed significance threshold of *p* < 0.05 was adopted for all tests.

#### 2.4.1 Preliminary tests

The Shapiro–Wilk test was first conducted to assess the normality of all continuous variables. Results indicated that all variables showed non-normal distributions, so descriptive statistics are reported as median with interquartile range (Q1–Q3) instead of mean and standard deviation. For between-group comparisons, the Mann–Whitney *U* test was used for two-group comparisons, and the Kruskal–Wallis *H* test was used for comparisons across three or more groups.

#### 2.4.2 Analytical procedures

First, Harman’s single-factor test was performed to evaluate potential common method bias. Descriptive statistics and Pearson correlation coefficients for all variables were then calculated.

Two regression analyses were conducted for distinct purposes. The first was a preliminary multiple regression (Table 1), which examined the independent overall associations between all core variables and emotional eating. Its main goal was to establish the statistical foundation for the subsequent indirect effect analysis. The second was a hierarchical regression for the indirect effect model (Table 2). This stepwise analysis tested the specific path coefficients needed to estimate direct and indirect effects in the sequential associative indirect effect model, following the procedures outlined by Hayes (2013).

**Table 1:** Multiple regression analysis of emotional eating.

Variables	Emotional eating				
	Unstandardized *b*	SE of *b*	95% CI for b	Standardized *β*	*t*
Self-objectification	0.02	0.03	[−0.04,0.08]	0.02	0.66
Social comparison	0.55	0.11	[0.33,0.78]	0.18	4.89***
Body satisfaction	−0.21	0.07	[−0.34,-0.08]	−0.12	−3.15**
BMI	−0.004	0.18	[−0.36,0.36]	−0.001	−0.02
*R*	0.25				
*R* ^2^	0.06				
*F*	13.65***				

*N* = 820. Model fit: *R* = 0.25, *R*^2^ = 0.06, adjusted *R*^2^ = 0.058. CI = confidence interval for unstandardized coefficient b on raw scale. *p < 0.05, **p < 0.01, ***p < 0.001.

**Table 2:** Regression analysis in the chain of intermediary model.

Variables	Social comparison			Body satisfaction			Emotional eating		
	*b*	*SE*	*t*	*b*	*SE*	*t*	*b*	*SE*	*t*
Grade	−0.04	0.07	−0.58	0.28	0.13	2.22*	0.41	0.24	1.72
Family structure	−0.40	0.31	−1.28	−0.42	0.53	−0.80	−0.07	1.00	−0.07
Place of household registration	0.67	0.29	2.33*	0.94	0.48	1.94	−0.36	0.93	−0.38
Relationship status	0.24	0.27	0.91	0.90	0.45	2.00*	−1.41	0.85	−1.65
BMI	0.16	0.06	2.91**	−0.81	0.09	−8.76***	−0.004	0.19	0.02
Self-objectification	0.09	0.01	9.62***	−0.10	0.02	−6.03***	0.02	0.03	0.75
Social comparison				−0.34	0.06	−5.81***	0.56	0.11	4.95***
Body satisfaction							−0.21	0.07	−3.10**
*R*	0.36			0.45			0.26		
*R* ^2^	0.13			0.20			0.07		
Adjusted *R*^2^	0.12			0.20			0.06		
*ΔR* ^2^	0.13			0.21			0.07		
*F*	20.28***			29.7***			7.64***		

*p < 0.05, **p < 0.01, ***p < 0.001.

All regression models were evaluated for key statistical assumptions before interpretation. Multicollinearity was assessed using variance inflation factor (VIF) values. Normality of residuals was examined with Q–Q plots, and homoscedasticity was verified using standardized residual plots.

Finally, statistical significance of all effects was determined using 5,000 bootstrap resamples to generate 95% bias-corrected confidence intervals (95% BC CIs). An effect was considered statistically significant if its 95% BC CI did not contain zero.

#### 2.4.3 Grouping strategy rationale

Median splits were used to explore the interactive effects of BMI and self-objectification for two methodologically justified reasons. For BMI grouping, the standard WHO clinical cut-offs (underweight<18.5, normal weight 18.5–24.9, overweight≥25) were unsuitable for this sample of Chinese female college students, who showed a narrow BMI distribution (median = 19.53, IQR = 18–21) with only 8.9% meeting the clinical criteria for overweight or obesity. Whereas median splits produced equal groups of 410 participants each and maximized power for this exploratory analysis. For self-objectification grouping, no universally accepted clinical or population-based cut-offs exist for the Trait Self-Objectification Questionnaire used in this study. Median splits are therefore the standard and widely accepted exploratory method for examining interactions between continuous variables in psychological research when no established cut-offs are available (Hayes, 2013).

### 2.5 Test of common method variance

Given that data were collected solely via questionnaires, common method variance (CMV) was a potential concern. We used Harman’s single-factor test to test the CMV. Results indicated seven factors with eigenvalues greater than 1. The initial eigenvalue of the first factor was 8.572, explaining 26.21% of the variance—below the established 40% critical threshold. Consequently, no severe common method bias was identified in this study.

## 3 Results

### 3.1 Descriptive statistics and sociodemographic differences

#### 3.1.1 Sociodemographic characteristics of the sample

Among the 820 Female college students (FCS) participants, 166 were freshmen (20.24%), 119 were sophomores (14.51%), 197 were juniors (24.02%), 181 were seniors (22.07%), and 157 were graduate students (19.15%); 559 participants (68.17%) were from rural areas, while 261 (31.83%) were from urban areas; 195 (23.78%) were only daughters, and 625 (76.22%) were not only daughters; 548 (66.83%) were single, and 272 (33.17%) were not single (Table 3).

**Table 3:** Sociodemographic characteristics and between-group differences in core variables.

Demographic variable	Grade					*H*	Hometown		*Z*	Family Structure		*Z*	Relationship Status		*Z*
	Freshmen (*n* = 166)	Sophomores (*n* = 119)	Juniors (*n* = 197)	Seniors (*n* = 181)	Graduate students (*n* = 157)		Rural areas (*n* = 559)	Urban areas (*n* = 261)		Only daughters (*n* = 195)	Non-only daughters (*n* = 625)		Single (*n* = 548)	Non-single (*n* = 272)	
Self-objectification	−7 (−18–5)	−5 (−15–5)	−5 (−15–5)	−5 (−15–7)	−7 (−15–5)	2.58	−7 (−15–5)	−3 (−15–7)	−1.21	−3 (−15–5)	−5 (−15–5)	−1.21	−7 (−15–5)	−3 (−15–7)	−1.60
Social comparison	13 (11–15)	14 (12–17)	14 (11–16)	13 (11–16)	13 (11–16)	7.58	13 (11–16)	14 (12–17)	−3.59**	14 (11–17)	13 (11–16)	−2.74***	13 (11–16)	14 (11–16)	−1.30
Body satisfaction	25 (21–29)	24 (21–30)	24 (21–29)	26 (22–30)	28 (23–33)	22.46***	25 (22–30)	26 (21–31)	−0.76	26 (21–31)	25 (21–30)	−0.55	24 (21–30)	26 (22–32)	−2.75**
Emotional eating	28 (22–36)	31 (25–39)	32 (24–41)	32 (25–39)	31 (25–40)	9.32	31 (25–39)	31 (24–40)	−0.02	31 (24–40)	31 (25–39)	−0.04	31 (25–39)	30 (23–39)	−1.10
BMI	20 (19–22)	19 (18–21)	20 (18–21)	19 (18–21)	20 (18–21)	9.43	20 (18–21)	20 (18–21)	−1.29	20 (18–21)	20 (18–21)	−1.11	20 (18–21)	19 (18–21)	−2.33*

H = Kruskal–Wallis *H* statistic (multi-group comparison); Z = Z statistic from Mann–Whitney *U* test (two-group comparison). Data are reported as median (interquartile range, Q1–Q3). *p < 0.05, **p < 0.01, ***p < 0.001.

*Post hoc* analyses based on Table 3 revealed key differences in core variables across demographic groups: Graduate students had significantly higher body satisfaction than freshmen, sophomores, and juniors. Urban FCS scored significantly higher in self-objectification and social comparison than rural ones. Only daughters exhibited higher social comparison levels than non-only daughters. Additionally, single FCS had significantly higher BMI and lower body satisfaction compared with non-single students.

#### 3.1.2 Between-group differences in core variables across sociodemographic factors

To explore the interactive effects of BMI and self-objectification on social comparison, body satisfaction, and emotional eating. Participants were divided into four groups based on the median values of body mass index (BMI = 19.53) and self-objectification scores (=−5.00). Table 4 indicate significant differences among FCS across groups, In social comparison, both G1 and G3 scored significantly lower than G2 and G4; Regarding body satisfaction, G1 scored significantly higher than G2, G3, and G4, while G2 and G3 both scored significantly higher than G4; In emotional eating, G4 scored significantly higher than G1 and G3.

**Table 4:** Differential analysis of groups.

Groups	Total	G1	G2	G3	G4	*H*
	*N* = 820	*n* = 224	*n* = 201	*n* = 213	*n* = 182	
Social comparison	13 (11–16)	12 (10–15)	15 (12–17)	13 (11–15)	14 (12–17)	70.68***
Body satisfaction	25 (21–30)	28 (24–33)	26 (22–32)	25 (21–29)	22 (18–25)	119.00***
Emotional eating	31 (25–39)	30 (24–37)	31 (25–40)	30 (24–38)	33 (27–41)	10.71*

G1–G4 represent the low BMI low self-objectification group, low BMI high self-objectification group, high BMI low self-objectification group, and high BMI high self-objectification group, respectively. Groups were divided by the median values of BMI (19.53) and self-objectification (−5.00). Between-group comparisons were performed using the Kruskal–Wallis *H* test. All values are presented as median (interquartile range, Q1–Q3). *p < 0.05, **p < 0.01, ***p < 0.001.

### 3.2 Correlation analysis among core variables

The prerequisite for cross-sectional indirect effect analysis is establishing correlations among the independent, dependent, and intermediate variables (Baron and Kenny, 1986). Therefore, bivariate correlation analyses were conducted on all study variables (Table 5). Results indicated that self-objectification had a moderate positive correlation with social comparison (*r* = 0.33, *p* < 0.01), a small-to-moderate negative correlation with body satisfaction (*r* = −0.26, *p* < 0.01), and a small positive correlation with emotional eating (*r* = 0.11, *p* < 0.01). Social comparison showed a small-to-moderate negative correlation with body satisfaction (*r* = −0.28, *p* < 0.01) and a small positive correlation with emotional eating (*r* = 0.22, *p* < 0.01). Body satisfaction had a small negative correlation with emotional eating (*r* = −0.17, *p* < 0.01).

**Table 5:** Correlation analysis.

Variable	1	2	3	4	5
1. Self-objectification	–				
2. Social comparison	0.33**	–			
3. Body satisfaction	−0.26**	−0.28**	–		
4. Emotional eating	0.11**	0.22**	−0.17**	–	
5. BMI	0.01	0.10**	−0.31**	0.05	–

All coefficients are Pearson correlation coefficients. Conventions: |*r*| < 0.1 = negligible, 0.1 ≤ |*r*| < 0.3 = small, 0.3 ≤ |*r*| < 0.5 = moderate, |*r*| ≥ 0.5 = large. Although variables showed mild non-normality, Pearson correlation was used due to the large sample size (*N* = 820), which provides robust estimates under central limit theorem. *p < 0.05, **p < 0.01, ***p < 0.001.

To preliminarily examine the independent overall associations of each core variable with emotional eating and validate the statistical foundation for subsequent indirect effect analysis, a multiple linear regression was conducted with emotional eating as the sole outcome variable (Table 1). Results showed that social comparison (*t* = 4.89, *p* < 0.001) significantly and positively predicted emotional eating, while body satisfaction (*t* = −3.15, *p* < 0.01) significantly and negatively predicted emotional eating. Self-objectification and BMI showed no significant independent associations with emotional eating in this preliminary model. The preliminary multiple regression model showed good fit (*F* = 13.65, *p* < 0.001, *R*^2^ = 0.06). No significant multicollinearity was detected, with all VIF values ranging from 1.01–1.12 (well below the 10 threshold).

### 3.3 Testing the sequential indirect effect of self-objectification and emotional eating

Self-objectification served as the independent variable, emotional eating as the dependent variable, and social comparison and body satisfaction as intermediate variables. Control variables included grade, family structure, hometown, relationship status, and BMI.

Table 2 presents the stepwise hierarchical regression results of the cross-sectional sequential indirect effect model, following the analytical procedure recommended by Hayes (2013). Unlike the preliminary regression in Table 1, this hierarchical analysis tests the specific path coefficients required to estimate the direct and indirect effects in the mediation framework.

The hierarchical regression model showed acceptable fit for all three steps. All VIF values remained below 1.20, confirming no multicollinearity issues. Residual assumptions were satisfied for all outcome variables. Regression analysis showed that: self-objectification positively predicted social comparison and negatively predicted body satisfaction; social comparison negatively predicted body satisfaction and positively predicted emotional eating; body satisfaction negatively predicted emotional eating (Table 2 for detailed statistics).

Results indicate that the indirect effect of social comparison on body satisfaction is significant, with a associative indirect effect size of 0.076, accounting for 76.45% of the total effect (0.100) between self-objectification and emotional eating.

Three significant indirect pathways were identified (all 95% confidence intervals excluded zero): (1) Self-objectification → Social comparison → Emotional eating (ab = 0.020, 49.90% of total effect); (2) Self-objectification → Body satisfaction → Emotional eating (ab = 0.020, 20.34% of total effect); and (3) Self-objectification → Social comparison → Body satisfaction → Emotional eating (ab = 0.006, 6.21% of total effect).

**Figure 1:**
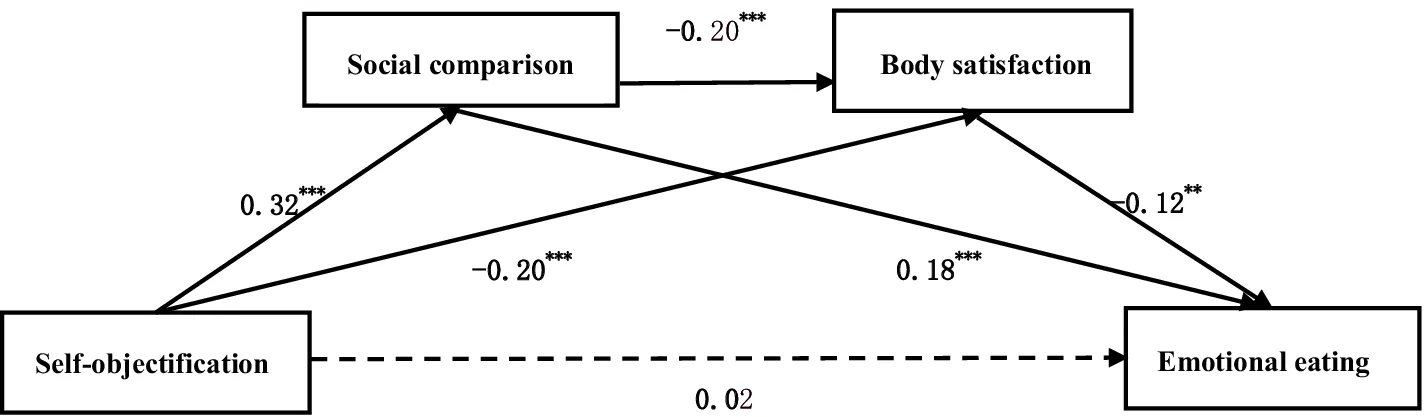
Cross-sectional associative indirect effect model diagram. Standardized effects (*β*) are presented in the figure. *p < 0.05, **p < 0.01, ***p < 0.001.

Comparisons of indirect effects across pathways revealed significant differences, which was supported by the 95% confidence intervals for C2 and C3 that did not include zero (Table 6). Specifically, the indirect effects of Path 1 (Self-objectification → Social comparison → Emotional eating) and Path 2 (Self-objectification → Body satisfaction → Emotional eating) were both significantly stronger than that of Path 3 (Self-objectification → Social comparison → Body satisfaction → Emotional eating). These findings further underpinned the construction of the study’s cross-sectional associative indirect effect model (Figure 1).

**Table 6:** Indirect effects analysis of self-objectification and emotional eating.

Pathway	Effect size	Bootstrap95%		Proportion mediated
		Lower limit	Upper limit	
Direct effects	0.024	−0.038	0.086	23.95%
Total indirect effects	0.076	0.049	0.105	76.45%
Ind1: Self-objectification → Social comparison → Emotional eating	0.050	0.026	0.075	49.90%
Ind2: Self-objectification → Body satisfaction → Emotional eating	0.020	0.007	0.023	20.34%
Ind3: Self-objectification → Social comparison → Body satisfaction → Emotional eating	0.006	0.002	0.011	6.21%
C1: Ind1–Ind2	0.030	−0.002	0.060	–
C2: Ind1–Ind3	0.044	0.020	0.069	–
C3: Ind2–Ind3	0.014	0.004	0.028	–

*N* = 820. Effects are unstandardized. 95% BC bootstrap CIs were calculated via 5,000 resamples. Ind1, Ind2, Ind3 represent three indirect pathways; C1–C3 denote pairwise comparisons of indirect effect values. Proportion mediated (%) = percentage of total effect explained by each pathway. Significance is confirmed when the 95% CI does not include zero.

## 4 Discussion

This study examined the association between self-objectification and emotional eating among 820 female college students in Yunnan, China. We systematically tested the parallel and sequential mediating roles of social comparison and body satisfaction.

The core finding is that self-objectification has no significant direct association with emotional eating. Instead, its total association is fully explained by three indirect pathways: the parallel pathway through social comparison, the parallel pathway through body satisfaction, and the sequential pathway through social comparison followed by body satisfaction. Existing research under objectification theory has focused largely on restrictive eating. Emotion-driven emotional eating remains understudied, especially among inland Chinese female college students. This study extends prior theoretical frameworks by unpacking these multiple mediating pathways in this population.

### 4.1 Interpretation of core indirect effects

#### 4.1.1 Non-significant direct effect of self-objectification on emotional eating

Objectification theory proposes that experiences of sexual objectification, such as being ogled or sexually harassed, contribute to the development of self-objectification (Zhao and Jiang, 2021). These experiences are also linked to negative outcomes including internalized shame, externalized shame, and disordered eating (Peng et al., 2017).

This pattern aligns with the view that self-objectification is associated with a greater likelihood of emotional eating. However, the direct association between self-objectification and emotional eating became non-significant after controlling for mediating variables and demographic covariates.

This finding challenges the simplistic assumption that self-objectification is directly linked to disordered eating behaviors. Instead, self-objectification functions as a distal correlate of emotional eating. Its association with emotional eating operates fully through intermediate cognitive and affective processes, namely social comparison and body satisfaction.

Female college students with higher self-objectification report more body-related negative emotions. These emotions are in turn associated with more dieting and binge eating behaviors (Güner and Aydın, 2022; Carr and Szymanski, 2011). This result refines the application of objectification theory to emotional eating. It highlights the need to include mediating variables to capture the complexity of this relationship.

#### 4.1.2 Parallel indirect effects of social comparison and body satisfaction

Social comparison had the largest separate indirect effect, accounting for 49.90% of the total association between self-objectification and emotional eating. This finding suggests that appearance-based social comparison is the most prominent pathway linking self-objectification to emotional eating in this sample.

Festinger (1954) conceptualized social comparison as an innate human tendency: an automatic cognitive process that reflects self-referential judgment. Extensive research shows that appearance-based social comparison contributes to negative self-perception and poor psychological adjustment (Wolff et al., 2000; Fardouly and Vartanian, 2015). Sociocultural theories also identify body image comparison as a key factor in the onset and progression of disordered eating (Annesi and Mareno, 2015).

Our results show that individuals with higher self-objectification engage in more frequent appearance comparisons in daily life. They rely on these comparisons to evaluate how closely they match societal beauty standards. These comparisons generate negative emotions such as anxiety, inferiority, and stress. Stress and negative affect are well-documented precursors to binge eating (Annesi, 2022). Thus, social comparison may operate as an intermediate variable that explains part of the association between self-objectification and emotional eating.

Body satisfaction also acted as a significant separate mediator, explaining 20.34% of the total effect. Emotional eating is a maladaptive coping response to negative emotional distress. It is consistently associated with higher perceived stress, anxiety, and depression (Geng, 2020).

Under narrow societal beauty standards, individuals with high self-objectification who do not meet these standards often experience body dissatisfaction, body disgust, and appearance anxiety. These negative body-related experiences are consistently linked to higher levels of emotional eating (Heatherton and Baumeister, 1991; Yehuda et al., 2000; Tang et al., 2019; Phillips et al., 2016).

As Heatherton and Baumeister (1991) proposed, this state of self-dissatisfaction typically causes significant emotional distress. Binge eating then emerges as a maladaptive coping mechanism that allows people to shift focus away from self-directed negative thoughts (Kaplan and Kaplan, 1957).

Our findings align with this theoretical framework. They suggest that body satisfaction may serve as an important bridging correlate in the associational pathway between self-objectification and emotional eating. Consistent with escape theory of self-awareness, individuals with lower body satisfaction may use eating as a coping tool to redirect attention away from painful self-evaluations and toward immediate sensory experiences. This result supports body dissatisfaction as a proximal correlate of emotional eating, and extends prior work by explicitly placing it within the self-objectification framework.

#### 4.1.3 Sequential indirect effect of social comparison and body satisfaction

A key contribution of this study is the empirical support for the sequential mediating pathway: self-objectification → social comparison → body satisfaction → emotional eating. This pathway accounted for 6.21% of the total association between self-objectification and emotional eating.

From a theoretical perspective, this chain pathway describes a sequential cognitive process linking self-objectification to emotional eating. First, higher self-objectification is associated with a greater tendency to engage in appearance-based social comparison. Second, more frequent appearance comparisons are associated with lower body satisfaction, as they may highlight gaps between one’s actual body and internalized beauty standards. Finally, lower body satisfaction is associated with more emotional eating as a way of regulating negative affect.

Theoretically, people who use maladaptive emotion regulation strategies for body-related negative affect may be more likely to engage in overeating. This pattern has been proposed to strengthen over time through a negative reinforcement mechanism. When binge eating temporarily reduces distress from appearance anxiety and body shame, people may become more likely to repeat this strategy during future emotional distress. Over time, this can develop into stable emotional eating patterns (Zheng et al., 2020).

Notably, the effect size of the sequential mediation pathway is relatively modest by two explicit criteria. First, in relative terms, this pathway accounted for only 6.21% of the total effect, which is substantially smaller than the two parallel indirect pathways (49.90 and 20.34%, respectively). Second, consistent with methodological conventions for serial mediation analysis (Hayes, 2013), indirect effects transmitted through two or more intermediate steps typically show stepwise attenuation. An indirect effect explaining less than 10% of the total effect is generally considered small in magnitude.

This pattern reflects progressive effect reduction across three consecutive psychological processes. Each step is shaped by multiple contextual and individual factors.

Beyond the relative size of specific pathways, the overall effect sizes observed in this study merit explicit discussion. Bivariate correlations between core study variables ranged from small to moderate (*r* = 0.11–0.33) according to Field’s (2020) conventional benchmarks. The full regression model predicting emotional eating explained 6–7% of the outcome variance (*R*^2^ = 0.06–0.07), representing a small-to-medium effect size.

As a distal sociocognitive construct, the association between self-objectification and specific eating behaviors operates through multi-step intermediate pathways. This structure leads to natural attenuation in observed effect sizes. Moreover, emotional eating is a complex behavioral phenotype shaped by a wide range of biological, genetic, familial, and environmental factors beyond body image-related pathways.

The relatively modest variance explained does not undermine the theoretical importance of the identified associations. Instead, it clarifies that the self-objectification framework captures one meaningful subset of the many contributors to emotional eating. The statistically significant pathways still carry practical implications for population-level intervention design.

Theoretically, this study provides initial empirical support for the complete sequential psychological chain linking self-objectification to emotional eating. It also clarifies underlying mediating mechanisms that have been underexplored in prior research.

Practically, our results indicate that addressing emotional eating among female college students requires a multi-level, sequential intervention approach within the broader context of sexual objectification. The rapid growth of online media has given women near-universal access to fashion and appearance-related content. This makes it nearly impossible to fully avoid potential experiences of online sexual objectification (Yi and Huang, 2021).

For this reason, interventions that target only self-objectification are unlikely to produce meaningful, lasting effects. Instead, interventions can interrupt this sequential pathway at multiple actionable points. These include reducing self-objectification-driven appearance comparisons, improving body satisfaction, and building adaptive emotion regulation skills. This multi-stage targeted approach offers greater flexibility and potential clinical utility than traditional single-target interventions. It may ultimately help break the cycle of using eating as the primary way to escape from body-related negative emotions.

### 4.2 Demographic correlates and the interactive effect of BMI and self-objectification

Several notable findings emerged from the demographic and BMI interaction analyses.

First, graduate students reported significantly higher body satisfaction than undergraduates. This aligns with prior work showing that older women have higher self-esteem and body satisfaction than younger women (Patel et al., 2025). It likely reflects greater identity consolidation and reduced appearance-related pressure as women progress through higher education.

Second, participants in romantic relationships had lower BMI and higher body satisfaction than single students. This finding supports prior research showing that women who receive more affectionate touch and report higher sexual satisfaction from their partners have greater body satisfaction (Pujols et al., 2010; Campbell et al., 2025).

Most importantly, we observed an interactive association between BMI and self-objectification. Among participants with higher BMI, those with low self-objectification had significantly higher body satisfaction than those with high self-objectification. Across all BMI levels, individuals with low self-objectification also reported less frequent appearance comparison and less emotional eating.

This pattern suggests that BMI alone is not a strong correlate of body image disturbance and disordered eating in this sample. Instead, its association with body satisfaction and emotional eating appears to vary according to individuals’ level of self-objectification. This finding adds to evidence questioning the primacy of BMI as a correlate of body dissatisfaction and disordered eating (Dakanalis et al., 2015; Mehak et al., 2018). It also highlights the underrecognized role of psychological factors in these outcomes.

One methodological limitation of this interaction analysis must be explicitly noted. We divided BMI groups using sample median values, rather than standard international clinical BMI cut-offs. Median split was selected to balance group sizes in this student sample with a narrow BMI distribution. However, this grouping method may reduce comparability with clinical and population research that uses established WHO BMI categories.

### 4.3 Theoretical and practical contributions

This study advances understanding of the relationship between self-objectification and emotional eating in three key ways.

First, it indicates that self-objectification is a distal correlate of emotional eating, rather than a direct independent predictor when cognitive and affective pathways are accounted for. This finding refines objectification theory by showing that the link between self-objectification and disordered eating is not uniform across different types of eating behaviors.

Second, support for the sequential mediating pathway reveals the hierarchical nature of the cognitive processes involved. Self-objectification is associated with more appearance-based social comparison, which is linked to lower body satisfaction, which in turn relates to more emotional eating as a coping strategy. This sequential model offers a more nuanced explanation than previous parallel mediation models.

Third, the finding that high self-objectification amplifies the negative association between high BMI and emotional eating has important theoretical implications. It suggests that psychological factors such as self-objectification may be more strongly associated with disordered eating patterns than objective body weight itself.

Practically, the identification of three distinct mediating pathways supports the development of multi-stage intervention programs. Interventions can target different points in the associational chain, from reducing self-objectification at the earliest stage to improving emotion regulation at the final stage. This multi-target approach may be more effective than traditional single-target interventions.

These findings also suggest that interventions for disordered eating in young women should not focus solely on weight management. Reducing self-objectification and fostering a functional body orientation may be promising intervention targets, especially for individuals with higher BMI. This is supported by the finding that lower self-objectification is associated with better body satisfaction and less emotional eating across BMI levels.

### 4.4 Limitations and future directions

This study has several limitations that should be acknowledged.

First, generalizability is limited. We recruited female college students from only two universities in Yunnan Province. We also did not examine potential influences of additional variables such as clinical diagnosis. Future studies should include diverse regions, age groups, and clinical populations to test the universality of the proposed model.

Second, definitive causal conclusions cannot be drawn. The cross-sectional design cannot establish the temporal order of variables or rule out reverse causality. Longitudinal cohort studies or experimental manipulations of self-objectification are needed to confirm causal relationships.

Third, the exploration of mediating mechanisms is incomplete. We only examined social comparison and body satisfaction as intermediate variables. Future research should investigate additional mediators and protective factors to further refine the theoretical model.

Fourth, several measurement and analytical biases exist. All data were collected via self-report questionnaires, which are susceptible to social desirability bias. BMI was calculated from self-reported height and weight rather than objective measurements. In addition, we used median split for BMI categorization to avoid extreme sample imbalance under clinical classification. Median split inevitably discards continuous data and carries a risk of spurious significance. Future research should use continuous BMI analysis or recruit larger samples covering a wider range of weight status. Future studies could also combine self-reports with experimental priming, ecological momentary assessment, and objective anthropometric measurements to improve overall data validity.

## 5 Conclusion

(1) Self-objectification positively correlates with emotional eating in Female college students (FCS) but has no significant direct effect. Social comparison and body satisfaction fully mediate this relationship through three pathways, with social comparison’s parallel mediation being the strongest (49.90% of total effect). (2) FCS with both higher BMI and higher self-objectification report higher levels of emotional eating. Core variables show significant demographic differences across grade, hometown and relationship status. (3) This study may help clarifies the underlying psychological mechanism, supporting targeted interventions for disordered eating. Future work should explore more mediators, optimize methods and expand sampling.

## Data Availability

The raw data supporting the conclusions of this article will be made available by the authors, without undue reservation.
